# Labial and Vaginal Microbiology: Effects of Extended Panty Liner Use

**DOI:** 10.1155/S1064744997000422

**Published:** 1997

**Authors:** M. A. Farage, N. A. Enane, S. Baldwin, R. W. Berg

**Affiliations:** Paper Products Division Procter & Gamble Company Winton Hill Technical Center 6100 Center Hill Avenue Cincinnati OH 45224 USA

## Abstract

*Objective:* The goals of this study were 1) to better define the labial microflora and 2) to evaluate whether extended non-menstrual use of panty liners would increase genital carriage of undesirable bacteria and predispose to infection.

*Methods:* Healthy female volunteers (224) were prospectively randomized into panty liner wear groups A (Always^®^ deodorant) and B (Always^®^ non-deodorant) and into a control group C (no panty liner wear) with instructions for non-menstrual ± menstrual use ≥5 h daily for 6 months. Selected aerobic bacteria were semiquantitatively cultured from the inner labial groove, the posterior fornix of the vagina, and the cervix pre-study and post-study. Used panty liners were quantitatively cultured, and vaginal secretions were examined by gas chromatography for fatty acid ratios as a measure of microbial flora shifts.

*Results:* At the pre-study, labial microflora in this study population contained significantly higher frequencies of *Staphylococcus*, coliforms, other gram-negative rods, and enterococci, and a decreased frequency of *Gardnerella vaginalis* relative to the vaginal microflora. After 6 months use of panty liners the frequencies (and densities) of the selected microorganisms in these two sites had not changed compared to controls, and fatty acid analyses of vaginal secretions gave no evidence of shifts in the microbial flora.

*Conclusions:* Frequencies of selected genital microflora were different for the labia compared to the vagina. No increased carriage of medically important species was detected for either site after 6 months of daily (average 7.8 h) panty liner use.


Infectious Diseases in Obstetrics and Gynecology 5:252–258 (1997)
© 1997 Wiley-Liss, Inc.

### Labial and Vaginal Microbiology: Effects of Extended Panty Liner Use

**M.A. Farage,* N.A. Enane, S. Baldwin, and R.W. Berg**
*Paper Products Division, Procter & Gamble Company, Cincinnati, OH*

ABSTRACT

*Objective:* The goals of this study were 1) to better define the labial microflora and 2) to evaluate whether extended non-menstrual use of panty liners would increase genital carriage of undesirable bacteria and predispose to infection.

*Methods:* Healthy female volunteers (224) were prospectively randomized into panty liner wear groups A (Always® deodorant) and B (Always® non-deodorant) and into a control group C (no panty liner wear) with instructions for non-menstrual ± menstrual use ≥5 h daily for 6 months. Selected aerobic bacteria were semiquantitatively cultured from the inner labial groove, the posterior fornix of the vagina, and the cervix pre-study and post-study. Used panty liners were quantitatively cultured, and vaginal secretions were examined by gas chromatography for fatty acid ratios as a measure of microbial flora shifts.

*Results:* At the pre-study, labial microflora in this study population contained significantly higher frequencies of *Staphylococcus*, coliforms, other gram-negative rods, and enterococci, and a decreased frequency of *Gardnerella vaginalis* relative to the vaginal microflora. After 6 months use of panty liners the frequencies (and densities) of the selected microorganisms in these two sites had not changed compared to controls, and fatty acid analyses of vaginal secretions gave no evidence of shifts in the microbial flora.

*Conclusions:* Frequencies of selected genital microflora were different for the labia compared to the vagina. No increased carriage of medically important species was detected for either site after 6 months of daily (average 7.8 h) panty liner use. Infect. Dis. Obstet. Gynecol. 5:252–258, 1997.
© 1997 Wiley-Liss, Inc.

KEY WORDS
genital; infection; sanitary pads; microflora

Panty liners are thin sanitary protection pads which often are used differently than thick absorbent catamenial pads. Panty liners are used to avoid panty soiling immediately pre- or post-menstruation or during menstruation (used concurrently with a tampon). Additionally, panty liners are used by many consumers during non-menstrual times to absorb female genital secretions, sweat, or urine (for women with light incontinence). Panty liners are worn in close contact with the genital mucosa, vulvar skin, and perineum, and could potentially affect these surfaces over time, through increased dryness, wetness, and/or occlusion, depending on the basis for use.[1] This could lead to changes in the normal physiology of the skin or to changes in the microbial flora which might predispose to infection.

At present, limited published data are available that address the microbial flora or skin and mucosal surface changes associated with sanitary pad use,[1–4] and the effects of panty liner use have not been published. The more extended periods of use of

*Correspondence to: Dr. Miranda A. Farage, Procter & Gamble Company, Winton Hill Technical Center, 6100 Center Hill Avenue, Cincinnati, OH 45224.

Clinical Study

Received 10 January 1997
Accepted 24 April 1997

panty liners, in particular, point to the need to evaluate possible microbial and physiological effects. This study was undertaken to 1) better define the microbiology of the labia and 2) to determine the effects of protracted use of deodorant and non-deodorant panty liners on the labial and vaginal microflora and on various gynecologic and dermatologic parameters. The study focused primarily on aerobic and facultative species which are potentially pathogenic or otherwise have a known association with vaginal, vulvar, or urinary tract infections. Detailed dermatologic and gynecologic observations are to be presented elsewhere.

### SUBJECTS AND METHODS

### Subjects

Healthy volunteers between the ages of 18 and 55 years were recruited for the study and provided a signed informed consent. Potential subjects were evaluated further by medical history and physical examination, and subjects were enrolled if deemed healthy by the principal investigator. Specific exclusions to the study were 1) a history of chronic vulvovaginal disorder, 2) current antibiotic therapy, and 3) present or anticipated pregnancy during the study. Participants were then randomized into test and control groups. The study design and informed consent form were approved by an institutional review board.

### Test Products

Two Always® (Procter & Gamble Company, Cincinnati, OH) panty liner products were tested (products A and B). Each was designed to absorb light menstrual flow and other vaginal discharge and was constructed of three layers: 1) a surface topsheet consisting of a perforated polyethylene film, 2) an absorbent core consisting of airlaid cellulosic tissue, and 3) a polyethylene backsheet film forming a moisture-impermeable barrier. Both panty liners were fastened to panties by an adhesive strip attached to the exterior of the backsheet. Panty liner A was a deodorant product containing a small amount of perfume added to the core close to the backsheet and panty liner B was a non-deodorant product.

### Study Design

This was a prospective, randomized, investigator-blinded, parallel study. A total of 224 women were randomly assigned to 3 mutually exclusive groups. Group A and group B used an Always® deodorant panty liner or an Always® non-deodorant panty liner, respectively, and group C did not wear a panty liner non-menstrually and served as a control group. Groups A and B were instructed to use only the test product provided to them for an average wear time of at least 5 h/day non-menstrually. All subjects were permitted to use their usual feminine hygiene products during menstruation, but otherwise were instructed not to use any new or untried feminine hygiene product aside from the study assignment.

Data were collected during 7 visits which included a pre-study visit (visit 0) and 6 further visits at monthly intervals (visits 1–6). Subjects began their panty liner assignment use after baseline data were obtained at visit 0. All subjects kept a diary and recorded physical activities, habits, and subjective assessment of wetness, dryness, and product comfort throughout the study.

### Examinations and Laboratory Tests

At visits 0 and 6, subjects underwent laboratory tests including blood chemistries, hematology, urinalysis, and Papanicolaou smear and a complete physical examination, gynecologic examination, and dermatologic assessment by a board-certified obstetrician-gynecologist. The gynecologic examination and the dermatologic assessment were repeated at each visit with normal and abnormal findings scored and recorded on standardized forms.

### Microbiological Tests

Samples were collected for microbiologic analyses during the pre-study (visit 0) and final (visit 6) pelvic examinations (each non-menstrual). Selected microorganisms were sought from swabs separately obtained from the inner labial groove, the endocervix, and the posterior fornix of the vagina. The media and methods for isolation and identification were as follows.

Cervical swabs were immediately plated onto New York City agar[5] and into 2SP transport medium for transport to the laboratory. *Neisseria gonorrhoeae* was identified as gram-negative diplococci which tested oxidase and coagglutination positive. *Chlamydia trachomatis* was identified from cell culture by formation of iodine-staining, intracytoplasmic inclusion bodies. *Mycoplasma hominis* and *Ure-*

TABLE 1. Bacterial enumeration grading scale

| Grade | No. of colonies in streak area 1st | 2nd | 3rd | 4th |
|---|---|---|---|---|
| 0 | Negative | | | |
| 1+ | ≤10 | | | |
| 2+ | >10 | ≤5 | | |
| 3+ | >10 | >5 | ≤5 | |
| 4+ | >10 | >5 | >5 | ≥1 |

*aplasma urealyticum* were identified by typical colonial morphology on New York City agar.

Swabs from the labial groove and posterior fornix were immediately transported to the laboratory and inoculated into media. Yeasts were grown on Sabouraud's agar, and *Candida albicans* was identified by a positive germ tube test. *Gardnerella vaginalis* was isolated from V agar[6] and identified by characteristic growth on plates (small colonies with diffuse beta-hemolysis) and Gram's stain (gram-negative to gram-variable coccobacillary forms). Staphylococci were isolated from sheep blood agar and mannitol salt agar and differentiated by coagulase testing: *Staphylococcus aureus* (+) and *Staphylococcus* sp. (–). Gram-negative rods were isolated on MacConkey plates and identified as coliforms (lactose fermenters), non-lactose fermenters, *Proteus* sp. (swarming growth), and *Pseudomonas* sp. (oxidase positive). Streptococci were isolated from sheep blood agar. Beta-hemolytic strains were designated group A (bacitracin sensitive), group B (CAMP positive), or group D (bile-esculin positive, includes enterococci). Alpha- or gamma-hemolytic strains were designated group D (bile-esculin positive, includes enterococci) or viridans (bile-esculin negative).

A semiquantitative measure of growth of the selected microorganisms from swabs was determined by noting the degree of growth in the various zones of plates streaked in a 4 quadrant fashion with progressive dilution. Enumeration of each selected colonial type was graded from 0 (no growth) through 4+; 1+, 2+, 3+, and 4+ referred to the highest streak zone that demonstrated light growth. The grading scale is shown in Table 1.

Additional microbiological tests performed only at visit 6 included quantitative culture for these selected microorganisms from a currently used panty liner worn to that visit. The panty liners were weighed (unused weight averages 1.5 g) and then vortexed in 10 ml of phosphate buffered saline; 5 ml was removed for preparation of serial 10-fold dilutions which were plated on the same media as used for labial swabs.

Also, at visit 6 only, the fatty acid composition of vaginal secretions was determined as a marker for the type of microflora in the vagina.[7] A normal lactobacilli-dominated flora has a succinate/lactate ratio of ≤0.4, whereas bacterial vaginosis is associated with a ratio of >0.4. A vaginal wash specimen was collected by introducing 3 ml of physiologic saline into the vagina, mixing this fluid with vaginal secretions with a swab, and removing by pipette a 1.0 ml sample from the vagina for analysis. This sample was placed in a test tube which was tightly closed, refrigerated, and transported to the laboratory within 24 h for analysis by gas liquid chromatography.[7]

### Statistical Analyses

Statistical comparisons between groups were performed using a distribution-free (non-parametric) analysis approach. The Kruskal-Wallis test was used throughout the study to provide the overall standard statistical hypothesis test $H_0$: $1_1 = 1_2 = 1_3$ vs. $H_A$: at least one inequality. Specific pairwise comparison of each treatment group with the control was done using the Wilcoxon rank sum test.[8] Additional multiple comparison techniques were evaluated, however, including linear contrast comparisons on the average ranks and approximate least significant difference (LSD) yardstick comparisons. Microbiologic and other data collected only at visit 0 and visit 6 were compared for groups separately for each visit, and differences at visit 6 compared to visit 0 were also analyzed relative to the control group for group comparison purposes.

### RESULTS

Of 224 women enrolled initially, 204 completed the study. No significant differences were found for age, race, birth control method, previously used menstrual or non-menstrual products, and history of vulvovaginal infection among test and control groups. These women were predominately between the ages of 26 and 35 years (mean 31 years) and premenopausal (93%). Most were Caucasian (88–92%, range among the groups), and almost all of the remainder were African-American. Most of the women were married and had one or no sex partner in the last 12 months. Among the groups,

>80% of women had not experienced any vulvovaginal infection within the past year, and histories among the remaining women generally revealed only one instance, usually a yeast infection. Sixty-seven percent had not previously used a non-menstrual product.

Women, on average, wore the test panty liner 7.8 h/day. For the gynecologic examinations and dermatologic assessments performed at monthly visits, no significant differences were observed in the parameters tested between groups A and B or between each of these groups and the control group C. Also, there were no statistical differences observed between visit 0 and visit 6 for physical examination, laboratory tests, or subject diary records.

### Microbiology of the Vagina and Labia

The frequencies and densities of selected microorganisms were compared for vaginal and labial sites (Table 2) in the total number of women (combined data from groups A, B, and C) at visit 0 prior to any study use of panty liners. Almost all of these selected types or species cultured were represented in both sites from the 224 women. Frequencies were significantly higher in the labial groove for a number of species, however, whereas *Gardnerella* was more common in the vagina (Table 2). For both sites, the isolation rates for *S. aureus* and group A *Streptococcus* were low, and *Pseudomonas* species were not found. The various species generally demonstrated light growth, between 1+ and 2+, in either site. These semiquantitative evaluations of density were not compared statistically.

### Pre-Study to Post-Study Effects on Microbial Flora

For the labial cultures, the isolation frequencies of the microbial type studies were compared for each of the test groups (A, B) against the control (C) pre-study (visit 0) and at visit 6. In three instances, statistically different frequencies (higher or lower in the test group) were discerned at pre-study, and in a single instance the frequencies were different at visit 6 (Table 3). None of these differences (Table 3) was suggestive of any clinically relevant, adverse finding. Importantly, when the isolation frequencies at visit 0 were compared to those of visit 6, the test groups showed no significant differences from the control group with respect to pre- vs. post-study frequencies. Likewise, the microbiology of the posterior fornix of the vagina revealed no differences in the pre-study to post-study comparisons against the control group, and for group comparisons at either visit there was only one instance of a significantly different frequency from pre-study values (Table 4).

**TABLE 2. Prestudy comparison of the frequencies and densities of selected aerobic microorganisms isolated from the posterior vaginal fornix versus the inner labial groove in 224 women**

| | Vagina[1] | | Labia[1] | |
|---|---|---|---|---|
| Microorganism | % Culture positive (density) | | % Culture positive (density) | |
| *Candida albicans* | 12.1 | (1.3) | 8.5 | (1.3) |
| Other yeasts | 3.1 | (1.2) | 2.7 | (1.4) |
| *Gardnerella vaginalis* | 12.9 | (2.2) | 4.0[2] | (1.5) |
| *Staphylococcus aureus* | 2.2 | (1.1) | 6.3[2] | (1.8) |
| *Staphylococcus* sp. | 35.3 | (1.2) | 87.1[2] | (1.9) |
| Coliforms | 17.0 | (1.7) | 37.9[2] | (1.3) |
| Gram-negative, nonlactose fermenters | 2.7 | (1.2) | 7.1[2] | (1.0) |
| *Proteus* sp. | 1.3 | (1.0) | 3.1 | (1.2) |
| *Pseudomonas* sp. | 0 | — | 0 | — |
| *Streptococcus*, Group A | 0.9 | (1.0) | 1.3 | (1.5) |
| *Streptococcus*, Group B | 8.9 | (1.8) | 10.3 | (1.7) |
| *Streptococcus*, Group D[3] | 19.6 | (1.5) | 30.8[2] | (1.9) |
| *Streptococcus*, beta-hemolytic, non A,B,D | 0 | — | 0.4 | (1.0) |
| *Streptococcus*, viridans | 15.2 | (1.8) | 19.6 | (1.7) |

[1]Percent of women culture-positive and semiquantitative, average density (growth on plate quadrant, scale 1+ to 4+) among women culture-positive for organism.
[2]Isolation frequency significantly different from vaginal site (Chi square, $p < 0.05$).
[3]Includes *Enterococcus*.

Cultures from cervical swabs revealed no differences when the same group comparisons were performed. None among the 224 women at visit 0 and the 204 women at visit 6 were culture positive for *N. gonorrhoeae*. Only one woman (control group) was positive for *C. trachomatis* (at visit 6). The frequencies observed for *U. urealyticum* were 27% (group A), 32% (group B), and 41% (group C) at visit 0 and, respectively, 33%, 24%, and 38% at visit 6. Frequencies of *M. hominis* ranged between 4% and 7% for the groups at visit 0 and between 2%

**TABLE 3. Comparison of the frequencies and densities pre-study and post-study of selected aerobic microorganisms isolated from the inner labial groove of women randomized to Always® deodorant (A), Always® non deodorant (B) panty liner products, and non-panty liner use control (C) groups**

| | Pre-study[1] Percent culture positive (density) | | | | | | Post-study[1] Percent culture positive (density) | | | | | |
|---|---|---|---|---|---|---|---|---|---|---|---|---|
| | A (n = 74) | | B (n = 75) | | C (n = 75) | | A (n = 64) | | B (n = 66) | | C (n = 74) | |
| Microorganisms | | | | | | | | | | | | |
| *Candida albicans* | 8 | (1.0) | 4[2] | (1.7) | 13 | (1.2) | 6 | (1.3) | 4 | (1.0) | 8 | (1.5) |
| Other yeasts | 3 | (1.0) | 1 | (2.0) | 4 | (1.3) | 3 | (1.0) | 3 | (1.0) | 4 | (1.3) |
| *Gardnerella vaginalis* | 4 | (1.3) | 2 | (2.0) | 5 | (1.3) | 2 | (2.0) | 0 | — | 3 | (1.0) |
| *Staphylococcus aureus* | 6 | (1.8) | 7 | (1.8) | 5 | (1.8) | 11 | (3.0) | 9 | (2.3) | 15 | (1.5) |
| *Staphylococcus* sp. | 89 | (1.9) | 83 | (2.0) | 89 | (1.9) | 81 | (1.6) | 83 | (1.8) | 81 | (1.5) |
| Coliforms | 43 | (1.1) | 39 | (1.4) | 32 | (1.1) | 41 | (1.2) | 48 | (1.5) | 39 | (1.3) |
| Gram-negative, non lactose fermenters | 12[2] | (1.0) | 8 | (1.0) | 1 | (1.0) | 16 | (1.3) | 11 | (1.0) | 8 | (1.2) |
| *Proteus* sp. | 3 | (1.0) | 4 | (1.0) | 2 | (1.5) | 5 | (1.3) | 8 | (1.8) | 5 | (2.5) |
| *Pseudomonas* sp. | 0 | — | 0 | — | 0 | — | 2 | (1.0) | 0 | — | 3 | (1.0) |
| *Streptococcus*, Group A | 3 | (1.0) | 1 | (2.0) | 0 | — | 0 | — | 0 | — | 0 | — |
| *Streptococcus*, Group B | 9 | (1.1) | 8 | (2.2) | 13 | (1.7) | 8 | (1.4) | 4[2] | (2.0) | 15 | (1.5) |
| *Streptococcus*, Group D[3] | 36 | (1.6) | 31 | (1.8) | 25 | (2.3) | 45 | (1.7) | 48 | (1.9) | 39 | (1.6) |
| *Streptococcus*, beta-hemolytic, non A,B,D | 0 | — | 0 | — | 1 | (1.0) | 1 | (1.0) | 0 | — | 0 | — |
| *Streptococcus*, viridans | 12[2] | (1.4) | 20 | (2.1) | 26 | (1.6) | 14 | (1.4) | 20 | (1.9) | 18 | (1.5) |

[1]Percent of women culture-positive and semiquantitative, average density (growth on plate quadrant, scale 1+ to 4+) among groups pre-study (Visit 0) prior to product wear and post-study (Visit 6) following 6 months non menstrual $\pm$ menstrual use of product (groups A and B).
[2]Isolation frequency significantly different from control for that visit (Wilcoxon Rank Sum Test, $p < 0.05$).
[3]Includes *Enterococcus*.

and 6% at visit 6. Among the culture positive women, either mycoplasmal species was present at a mean density of 1+ plate growth.

### Assay for Microbial Fatty Acid End-Products

Among the 204 women tested at visit 6, the percent of women with normal succinate/lactate ratios was virtually the same for the deodorant and non-deodorant panty liner users and the controls (84–85%). Abnormal ratios (>0.4) were present in 13% (A), 6% (B), and 8% (C), and end-products were undetectable in the remainder of the women. There were no significant differences compared to the control group.

### Microbial Load in Used Panty Liners

The frequencies and densities of the selected microbes were not different in used deodorant compared to used non-deodorant panty liners which were removed and analyzed at visit 6 (Table 5). These quantitative counts (reported per gram of used panty liner) also did not reveal any apparent increased growth of one potentially pathogenic organism relative to other species. The frequencies in used panty liners (Table 5) were remarkably similar to the frequencies from labial cultures at visit 6 (Table 3) for most of the organisms. Microbial densities (labial vs. pad) cannot be compared because of confounding variables of the different sample sizes and methods used.

### DISCUSSION

Previous reports of the microflora of the external genitalia generally have emphasized periurethral sites and urinary tract infections. In this study, the labial colonization pattern was established for selected microbes and compared to vaginal colonization. The colonization pattern was broadly similar. The labia, however, harbored certain species at higher frequencies compared to the vagina. Although these sites each have a different milieu, the increased frequencies probably were also related to the closer proximity of the labia to the perineum in the case of intestinal tract-associated microbes such as coliforms and enterococci and to a strong association with the skin in the case of the coagulase negative, normal skin flora staphylococci.

Colonization with *Gardnerella* was at a lower frequency on the labia than in the vagina, which is its virtually unique habitat. The incidence of *Gardner-*

TABLE 4. Comparison of the frequencies and densities pre-study and post-study of selected aerobic microorganisms isolated from the posterior vaginal fornix of women randomized to Always® deodorant (A), Always® non deodorant (B) panty liner products, and non-panty liner use control (C) groups

| | Pre-study[1] | | | | | | Post-study[1] | | | | | |
|---|---|---|---|---|---|---|---|---|---|---|---|---|
| | Percent culture positive (density) | | | | | | Percent culture positive (density) | | | | | |
| | A (n = 74) | | B (n = 75) | | C (n = 75) | | A (n = 64) | | B (n = 66) | | C (n = 74) | |
| Microorganism | | | | | | | | | | | | |
| *Candida albicans* | 8[2] | (1.2) | 12 | (1.8) | 16 | (1.2) | 6 | (1.5) | 9 | (1.2) | 7 | (1.0) |
| Other yeasts | 3 | (1.0) | 2 | (1.5) | 4 | (1.0) | 2 | (1.0) | 2 | (1.0) | 5 | (1.3) |
| *Gardnerella vaginalis* | 15 | (2.5) | 11 | (2.0) | 13 | (2.5) | 8 | (2.0) | 9 | (2.2) | 13 | (2.2) |
| *Staphylococcus aureus* | 1 | (1.0) | 4 | (1.3) | 1 | (1.0) | 8 | (2.0) | 2 | (1.0) | 5 | (1.0) |
| *Staphylococcus* sp. | 30 | (1.3) | 36 | (1.2) | 40 | (1.2) | 36 | (1.2) | 39 | (1.1) | 36 | (1.2) |
| Coliforms | 15[2] | (1.5) | 16 | (1.8) | 20 | (1.9) | 11 | (1.6) | 21 | (1.6) | 19 | (1.6) |
| Gram-negative, non lactose fermenters | 6 | (1.5) | 3 | (1.0) | 0 | — | 3 | (1.5) | 2 | (2.0) | 1 | (1.0) |
| *Proteus* sp. | 1 | (1.0) | 1 | (1.0) | 1 | (1.0) | 2 | (1.0) | 3 | (2.5) | 3 | (3.5) |
| *Pseudomonas* sp. | 0 | — | 0 | — | 0 | — | 0 | — | 0 | — | 0 | — |
| *Streptococcus*, Group A | 1 | (1.0) | 1 | (1.0) | 0 | — | 0 | — | 0 | — | 0 | — |
| *Streptococcus*, Group B | 6 | (1.6) | 7 | (2.2) | 12 | (1.7) | 3 | (2.0) | 3 | (1.5) | 11 | (2.1) |
| *Streptococcus*, Group D[3] | 16 | (1.2) | 19 | (1.3) | 24 | (1.9) | 14 | (1.4) | 21 | (1.2) | 11 | (1.7) |
| *Streptococcus*, beta-hemolytic, non A,B,D | 0 | — | 0 | — | 0 | — | 0 | — | 0 | — | 0 | — |
| *Streptococcus*, viridans | 17 | (1.8) | 16 | (1.7) | 12 | (1.9) | 8 | (1.4) | 14 | (1.2) | 14 | (1.4) |

[1]Percent of women culture-positive and semiquantitative, average density (growth on plate quadrant, scale 1+ to 4+) among groups pre-study (Visit 0) prior to product wear and post-study (Visit 6) following 6 months non menstrual ± menstrual use of product (groups A and B).
[2]Isolation frequency significantly different from control for that visit (Wilcoxon Rank Sum Test, $p < 0.05$).
[3]Includes *Enterococcus*.

*ella* in the vagina was somewhat lower in the present study, however, compared to published reports on women with a normal vaginal examination,[7,9–12] and detection might have been enhanced with truly selective media.[7,11,12] Among women with bacterial vaginosis, *Gardnerella* is present at nearly 100% incidence and at a high density.[7,9–12].

Aside from *Gardnerella*, the frequencies in the vagina of the selected aerobic and facultative vaginal microbes were not different, in general, from ranges reported for normal women in other studies.[10,11] Facultative lactobacilli were not sought, but lactobacilli are the predominant flora among women with normal vaginal findings.

The question of whether the patterns of more constant daily wear of panty liners by some consumers would increase the carriage of medically important labial and vaginal species over time was addressed by incorporating a protracted 6 month test period for wear into the present study. Theoretical concerns such as epithelial irritation leading to predisposition to increased carriage of *Candida* or frank yeast infections, or an increased labial or vaginal colonization with perineal type organisms (coliforms, other gram-negative rods, or enterococci) through transfer on the pad to the vulvar area, were not borne out. No changes were detected in the frequencies or densities of the selected labial, vaginal, and cervical microbes which would suggest any adverse clinical outcome. Likewise, the succinate/lactate ratios of vaginal wash samples, determined post-study as a marker for normalcy of the microflora, indicated a low rate of bacterial vaginosis[2] among both test and control groups. Cultures of used panty liners did not reveal any increased frequency or excessive growth of any potential pathogen in the panty liner itself during wear.

Published data on periodic menstrual use of the thicker catamenial pads (sanitary napkins) also suggest only limited effects on vaginal microbiology. In a cross-sectional study,[3] a higher incidence of coliforms in the vagina was associated with reported menstrual use of sanitary napkins compared to tampons. In another study, exclusive napkin users had a significantly higher rate of isolation of *Eubacterium* species, a lower rate of *Lactobacillus* species, and lower counts of coagulase negative staphylococci from the vagina, apparent only during the menstrual period, compared to tampon users in a total of 35 subjects.[4]

In summary, these present data increase our understanding of the microbiology of the labia, in par-

TABLE 5. Comparison of the frequencies and densities of selected aerobic microorganisms isolated from used Always® deodorant versus used Always® nondeodorant panty liners

| Microorganism | Deodorant[1] Percent culture positive (density) (n = 64) | | Non deodorant[1] Percent culture positive (density) (n = 66) | |
|---|---|---|---|---|
| *Candida albicans* | 5 | (6.62) | 2 | (6.53) |
| Other yeasts, not *Candida* | 5 | (7.23) | 2 | (6.11) |
| *Candida* sp. | 0 | — | 2 | (5.0) |
| *Gardnerella vaginalis* | 0 | — | 2 | (7.08) |
| *Staphylococcus aureus* | 13 | (6.80) | 9 | (6.59) |
| *Staphylococcus* sp. | 95 | (7.11) | 98 | (7.08) |
| Coliforms | 39 | (5.82) | 29 | (6.41) |
| Gram-negative, non lactose fermenters | 9 | (5.36) | 9 | (4.54) |
| *Proteus* sp. | 3 | (5.11) | 8 | (5.96) |
| *Pseudomonas* sp. | 0 | — | 0 | — |
| *Streptococcus*, Group A | 0 | — | 0 | — |
| *Streptococccus*, Group B | 2 | (6.26) | 2 | (5.78) |
| *Streptococcus*, Group D[2] | 39 | (6.72) | 41 | (6.67) |
| *Streptococcus*, beta-hemolytic, non A,B,D | 2 | (7.36) | 0 | — |
| *Streptococcus*, viridans | 22 | (6.34) | 23.3 | (6.46) |

[1]Percent of pads culture-positive and quantitative counts (mean $\log_{10}$ colony forming units per gram of panty liner) from pads which were culture-positive for the organism.
[2]Includes *Enterococcus*.

ticular, and indicate that extended use of deodorant or non-deodorant panty liners of this type is unlikely to adversely affect the labial or vaginal microflora.

### ACKNOWLEDGMENTS

The authors thank M. Milligan for statistical analysis, and G. Hill, Ph.D., and J. Huggins, Ph.D., for assistance in preparation of the manuscript.

### REFERENCES

1. Hanke-Baier P, Johannigmann F, Levin RJ, Wagner G: Evaluation of vaginal and perineal area during the use of external sanitary protection throughout the menstrual cycle. Acta Obstet Gynecol Scand 73:486–491, 1994.
2. Wilhelm D, Elsner P, Pine HL, Maibach HI: Evaluation of vulvar irritancy potential of a menstrual pad containing sodium bicarbonate in short-term application. J Reprod Med 36:556–560, 1991.
3. Watt B, Goldacre MJ, Loudon N, Annat DJ, Harris RI, Vessey MP: Prevalence of bacteria in the vagina of normal young women. Br J Obstet Gynaecol 88:588–595, 1981.
4. Chow AW, Bartlett KH: Sequential assessment of vaginal microflora in healthy women randomly assigned to tampon or napkin use. Rev Infect Dis 11(Suppl 1):S68–S73, 1989.
5. Faur YC, Weisburd MH, Wilson ME, May PS: A new medium for the isolation of pathogenic *Neisseria* (NYC medium). I. Formulation and comparisons with standard media. Health Lab Sci 10:44–52, 1973.
6. Greenwood JR, Pickett MJ, Martin WJ, Mack EG: *Haemophilus vaginalis (Corynebacterium vaginalis)* method for isolation and rapid biochemical identification. Health Lab Sci 14:102–106, 1977.
7. Spiegel CA, Amsel R, Eschenbach D, Schoenkneckt R, Holmes KK: Anaerobic bacteria in nonspecific vaginitis. N Engl J Med 303:601–607, 1980.
8. Hollander M, Wolfe DA: Nonparametric Statistical Methods. New York: John Wiley & Sons, 1973.
9. Amsel R, Totten PA, Spiegel CA, Chen KCS, Eschenbach D, Holmes KK: Nonspecific vaginitis: Diagnostic criteria and microbial and epidemiologic associations. Am J Med 74:14–22, 1983.
10. Hillier SL, Krohn MA, Rabe LK, Klebanoff SJ, Eschenbach DA: The normal vaginal flora, $H_2O_2$-producing lactobacilli, and bacterial vaginosis in pregnant women. Clin Infect Dis 16(Suppl 4):S273–S281, 1993.
11. Hill GB, Eschenbach DA, Holmes KK: Bacteriology of the vagina. Scand J Urol Nephrol Suppl 86:23–39, 1985.
12. Hill GB: The microbiology of bacterial vaginosis. Am J Obstet Gynecol 169:450–454, 1993.
